# Utility of Healthcare System-Based Interventions in Improving the Uptake of Influenza Vaccination in Healthcare Workers at Long-Term Care Facilities: A Systematic Review

**DOI:** 10.3390/vaccines8020165

**Published:** 2020-04-05

**Authors:** Angela Bechini, Chiara Lorini, Patrizio Zanobini, Francesco Mandò Tacconi, Sara Boccalini, Maddalena Grazzini, Paolo Bonanni, Guglielmo Bonaccorsi

**Affiliations:** 1 Department of Health Sciences, University of Florence, Viale GB Morgagni 48, 50134 Florence, Italy; angela.bechini@unifi.it (A.B.); chiara.lorini@unifi.it (C.L.); sara.boccalini@unifi.it (S.B.); paolo.bonanni@unifi.it (P.B.); guglielmo.bonaccorsi@unifi.it (G.B.); 2 Nuovo Ospedale delle Apuane, North-West Tuscany LHU, Via Enrico Mattei, 21, 54100 Massa, Italy; francesco.mandotacconi@uslnordovest.toscana.it; 3 Careggi, University Hospital, Largo G. Alessandro Brambilla, 3, 50134 Florence, Italy; grazzinim@aou-careggi.toscana.it

**Keywords:** healthcare professionals, healthcare workers (HCWs), influenza vaccination, long-term care facilities (LTCFs), nursing homes, staff, utility, vaccination coverage

## Abstract

Healthcare workers (HCWs) in long-term care facilities (LTCFs) can represent a source of influenza infection for the elderly. While flu vaccination coverage (VC) is satisfactory in the elderly, HCWs are less likely to be vaccinated. There is no definitive evidence on which types of healthcare system-based interventions at LTCFs would be more useful in improving the vaccination uptake among HCWs. We performed a systematic review in different databases (Pubmed, Cochrane Database of Systematic Reviews, Health Evidence, Web of Science, Cinahl) to provide a synthesis of the available studies on this topic. Among the 1177 articles screened by their titles and abstracts, 27 were included in this review. Most of the studies reported multiple interventions addressed to improve access to vaccination, eliminate individual barriers, or introduce policy interventions. As expected, mandatory vaccinations seem to be the most useful intervention to increase the vaccination uptake in HCWs. However, our study suggests that better results in the vaccination uptake in HCWs were obtained by combining interventions in different areas. Educational campaigns alone could not have an impact on vaccination coverage. LTCFs represent an ideal setting to perform preventive multi-approach interventions for the epidemiological transition toward aging and chronicity.

## 1. Introduction

The burden of disease due to seasonal influenza is high in terms of morbidity and mortality (250,000–500,000 deaths worldwide) every year. Recent estimates suggest that up to 650,000 people die of respiratory diseases linked with seasonal flu every year, with most deaths occurring among people aged over 75 years [1,2,3].

The risk groups for influenza include subjects having an increased risk of exposure to influenza viruses as well as those having a high risk of developing severe disease and complications with a high risk of dying if not hospitalized. Healthcare workers (HCWs) belong to the first group, as they are more exposed to influenza than the general population, while elderly people belong to the second group. Generally, the elderly population is at high risk of complications due to respiratory infections. This is shown by the high mortality rates in this population during the influenza seasons around the world [4,5,6,7].

Vaccination is the main public health intervention to prevent influenza. It can prevent significant annual morbidity and mortality, especially in healthcare settings [8,9]. Internationally, all available vaccines for the control of seasonal influenza are safe and effective, and their appropriated use to confer the highest protection to each individual [10,11,12]. Moreover, influenza immunization is a priority in countries where an aging population is on the rise [13,14,15,16,17,18], even though the effectiveness of influenza vaccination of HCWs in preventing influenza in the elderly is not conclusive [19].

HCWs working at long-term care facilities (LTCFs) are in close contact with the elderly and can represent a source of infection for those fragile subjects. Several studies show that healthcare professionals work at those facilities even after contracting the flu [8,20,21,22,23,24,25,26].

Actually, influenza outbreaks in LTCFs are very common as they occur in as many as 50% of such facilities each year [27].

According to some authors, there is a positive correlation among increasing vaccination rates in HCWs and a decrease in influenza diseases in the elderly [28,29,30,31,32,33,34,35]. Salgado et al. have shown that improving the vaccine acceptance rate of clinicians can prevent nosocomial influenza [36] Moreover, it could be a cost-effective intervention [37,38].

Nevertheless, while vaccination coverage rates are satisfactory in the elderly population at the hospital or healthcare settings, HCWs are less likely to be vaccinated, regardless of international recommendations [4,39,40]. Additionally, influenza vaccination coverage among healthcare personnel working in LTCFs is consistently lower than those working in all other healthcare settings (i.e. hospitals, ambulatory care, physician’s office) [41,42]. Most European member states recommend influenza vaccinations for all HCWs, while some of them recommend vaccination only for some HCW categories (e.g., staff with close contact with patients, staff with no patient contact but contact with potentially contaminated material, or social care staff directly involved in frontline patient care) [41].

Even in the context of specific recommendations, the healthcare system is the cornerstone of vaccination promotion. Since “the health system consists of all organizations, people, and actions whose primary intent is to promote, restore, or maintain health” and “healthcare providers are institutions or individuals providing healthcare services,” LTCFs are part of the healthcare system [43].

Interventions based on the healthcare system involve the use of coordinated activities that are implemented primarily in healthcare settings. Generally, these interventions require organizational changes by considering the healthcare system as a whole [44]. Moreover, changes in one part of a system is estimated to influence the other parts and, therefore, preventive interventions should focus on the entire process of patient care.

Healthcare system-based strategies may be included in the quality improvement of care and can foresee knowledge management (e.g., training of staff) and redesign of professional roles (e.g., lead professional for vaccination promotion) [45].

There is still no definitive evidence on which types of healthcare system-based interventions at LTCFs are more useful in improving the vaccination uptake among HCWs. Therefore, the aim of this systematic review was to provide a synthesis of the available studies regarding this topic.

## 2. Materials and Methods

### 2.1. Search Strategy

One of the authors conducted a database search in the following databanks: Pubmed, Cochrane Database of Systematic Reviews, Health Evidence, Web of Science, and Cinahl. The last search was completed on 31 January, 2020. No temporal limits were applied. Only articles in written English were included.

Research manuscripts reporting large datasets that are deposited in a publicly available database should specify where the data have been deposited and provide the relevant accession numbers. If the accession numbers have not yet been obtained at the time of submission, please state that they will be provided during review. They must be provided prior to publication.

Intervening studies involving animals or humans and other studies requiring ethical approval must list the authority that provided approval and the corresponding ethical approval code. We followed the search strategy in keeping with the PICO scheme—Cochrane Handbook for Systematic Reviews of Interventions [46]—which is summarized in the following question: “Are healthcare system-based interventions useful in increasing the uptake of influenza vaccination in healthcare workers in long-term facilities?”

The search strategy followed on Pubmed and Web of Science was prepared by selecting groups of keywords for each part of the PICO scheme. Each group was combined with others through the Boolean operator AND. For Cinhal, Health evidence, and Cochrane library, a simplified search strategy was performed (Box S1). All electronic database search results were combined in Endnote and duplicate records were removed.

Only primary studies, systematic reviews, and meta-analyses were considered. Among them, we selected those which were considered relevant in terms of interventions, populations, and outcomes defined in the research question.

Three reviewers independently screened all the titles, abstracts, and full texts to assess the studies that met the selection criteria. In case of any discrepancies or disagreements during the selection phase, a fourth researcher was consulted.

The Preferred Reporting Items for Systematic Reviews and Meta-Analyses (PRISMA) flow diagram guidance was used to display studies that were identified by the database search and met the inclusion and exclusion criteria [47].

### 2.2. Definition of HCWs, LTCFs, Type of Intervention

HCWs were defined as the workforce in charge of a healthcare facility who directly (doctors and nurses) or indirectly (ancillary or technical staff, pharmacists, cleaners…) delivers healthcare services to the residents.

There is no internationally recognized standard definition for LTCFs. According to European Centre for Disease Prevention and Control (ECDC), the term ‘long-term care services’ refers to the organization and delivery of a broad range of services and assistance to people who are limited in their ability to function independently on a daily basis. Generally, they respond to both health and social needs [48].

In our study, LTCFs were defined as any residential organization that houses older adults or elderly individuals with the assistance of medical and nursing staff.

For the inclusion in the final list of selected papers, healthcare system-based interventions can be grouped into one of the following three types of approaches/areas.

(a) Interventions aimed at enhancing access to vaccination services (expanded access in healthcare settings, reduced or free vaccination costs, on-site vaccination, etc.),
(b) Interventions to eliminate individual barriers (reminder and recall systems, education, incentives, etc.),
(c) Policy/leadership interventions directed to mandate vaccination coverage in HCWs (mandatory vaccination as a condition for employment, severe restrictions for unvaccinated HCWs, declination forms, etc.).

The investigated outcome was an increasing rate of influenza vaccination in HCWs in any dose, preparation, or time schedule compared to other kinds of intervention or no intervention. We analysed the effects of interventions on both immediate and long-term changes in influenza vaccination rates. We excluded studies reporting only serological outcomes if they did not include or report either an intervention to increase vaccination rates or an outcome of vaccination rates.

### 2.3. Quality Assessment

Papers were assessed using the Effective Public Health Practice Project’s (EPHPP) quality assessment tool for quantitative studies [49]. Studies of weak quality were excluded from the narrative data synthesis.

## 3. Results

### 3.1. General Description of the Collected Studies

**Figure 1:**
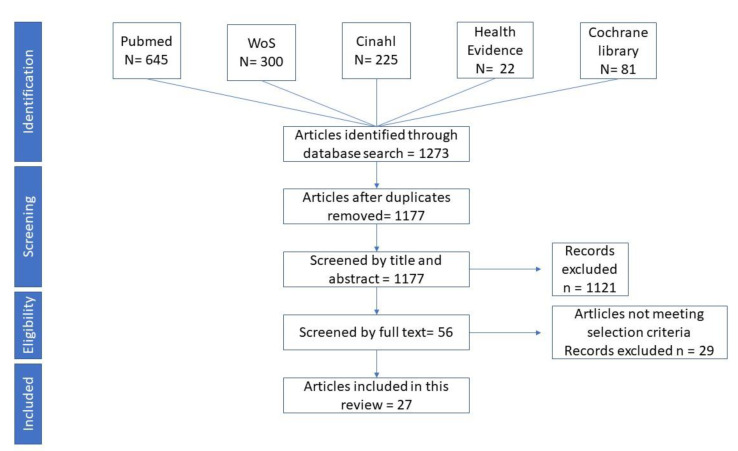
Preferred Reporting Items for Systematic Reviews and Meta-Analyses (PRISMA) flow diagram.

The literature search and selection of articles has been described in Figure 1. Among the 1177 articles screened by their titles and abstracts, 27 papers were included in the current study. They were all primary studies that met the inclusion criteria of the PICO model and, therefore, was included in our review.

Table 1 shows the list of the healthcare system-based interventions performed in the studies included in the final synthesis. They were classified into three areas, as previously described in the ‘Materials and Methods’ section.

Tables S1 and S2 report the synthesis of the included studies. Seven were randomized controlled trials [50,51,52,53,54,55,56], six were pre-post surveys [57,58,59,60,61,62], one assessed the vaccination rate trend in accordance with the types of interventions [63], 11 were cross sectional studies [64,65,66,67,68,69,70,71,72,73,74], and two were interrupted time series studies [75,76]. The majority (52%) of the selected studies were performed in the USA, which was followed by Europe (33%), Canada (11%), and Australia (4%). The number of facilities involved varied between one to 2303 facilities. Regarding the target population considered, 15 studies (56%) engaged not only health professionals, but all the people working at the facility.

With the EPHPP quality assessment tool [49], seven out of the 27 studies included in the current review were assessed as works of ‘strong’ quality [50,51,52,53,54,55,56], five papers of ‘moderate’ quality [58,63,72,74,75], and the remaining 15 works as ‘weak’ quality [56,59,60,61,62,64,65,66,67,68,69,70,71,73,76]. Results of the application of the EPHPP quality assessment tool are reported in Tables S1 and S2. Generally, most of the studies reported multiple interventions addressed to improve access to vaccination, to eliminate individual barriers, or to introduce policy interventions aimed at improving vaccination uptakes.

In 26 studies, the adopted interventions were associated with a higher vaccination rate or an increased level of vaccine uptake. In just one study [65], no significant association was found between the financing of influenza vaccination for HCWs and/or a vaccination promotion campaign and an increase in the vaccination rate. Two studies reported the effect of the interventions proposed as part of the quality improvement process [61,76].

### 3.2. Type of Interventions

Most of the studies examined the effect of multiple interventions with simultaneous components belonging to multiple areas presented in Table 1. Seventeen studies investigated the effect of interventions to improve access to vaccination. Twelve studies evaluated the effect of the availability of vaccination on worksites (1a) [51,53,55,57,63,67,68,69,70,71,74,76], and four of the vaccinations were offered during day and night shifts (1b) [53,69,71,76] while four of the organized vaccination events (1c) [54,55,62,63], and eleven of the vaccinations were offered free of charge or at a reduced cost (1d) [51,52,57,60,64,65,66,67,68,73,74].

Twenty-three studies evaluated the effect of interventions to eliminate individual barriers (attitudes/opinions). Three studies evaluated the effect of “identification of the individual and context barriers to vaccination” (2a), [54,61,76] 21 of “providing in-service educational or seminars educational sessions or counselling sessions for staff” (2b) [51,52,54,55,56,58,59,60,61,62,63,65,66,67,69,71,73,74,75,76], six of “displaying educational written materials” (2c) [51,52,53,55,56,70], two of “offering incentives to vaccinated healthcare workers” (2d) [61,64], and three “facilities providing reminders to staff to be immunized” (2e) [67,70,75].

Eighteen studies examined policy/leadership interventions that lead to vaccination. Five “have a policy on immunization” (3a) [53,60,67,69,72], five have a “mandatory vaccination policy” (3b) [57,63,64,71,74], three have “facilities recommending influenza immunization for staff” (3c) [51,67,70], three “require completion of declination forms” (3d) [61,66,75], five “identify lead personnel to arrange for vaccination sessions and vaccination promotion” (3e) [51,53,58,59,74], one “facilitates support by experts” (3f) [50], five “document vaccination status of staff and share feedback and goals” (3g) [59,60,67,71,76], one arranges “patient restriction for sick employees” (3h) [64], two consider the “vaccination rate as a target for quality improvement” (3j) [61,76], and three refer to “leadership involvement” (3k) [59,61,76].

### 3.3. Studies with Interventions Related to One Area

Five studies evaluated the effect of interventions related to only one area [50,56,58,68,72]. Two [56,58] evaluated the effect of interventions to eliminate individual barriers, while two [50,72] more evaluated the effect of policy interventions and one [68] more evaluated the effect of interventions to increase access to vaccination. One study [50] was of strong quality and evaluated the effect of ‘facilitation support by experts’ demonstrating an increase in the vaccination rate (at the facility-level) in the intervention group, from 65% at baseline (2008–2009) to 87% (2011–2012). On the contrary, in the control group, the vaccination rate ranged from 72–92% at baseline (2008–2009) to 67–80% (2011–2012). Two studies [58,72] were of moderate quality. The first study [58] assessed the impact of a training programme. Facilities participating in a single collaborative training programme improved the immunization rates modestly from 39.2% (2002) to 50.1% (2003), while facilities not participating saw decreases in such rates. The second study [72] found that the vaccination rate was 22% among care homes without a policy on staff influenza immunization, compared with 42% among care homes that did have such a policy (*p* = 0.01). However, it was not possible to establish what kind of policies were in use.

### 3.4. Studies with Interventions Related to Two Areas

Ten studies [52,54,55,57,59,61,62,65,73,75] evaluated the effect of interventions related to two areas taken together. Seven studies [52,54,55,62,65,73,75] applied a combination of better access to vaccination and reduction of individual barriers. One [59] performed leadership interventions and reduction of individual barriers, and two [57,61] combined easy access and leadership interventions. Three were assessed of strong quality [52,54,57]. In the first study [52], free vaccination was offered by general practitioners to both intervention and control groups. However, the intervention group was visited by a public health nurse, who performed an educational campaign while disseminating promotional materials and informing staff where they could obtain vaccination free of charge. No significant difference in vaccination rates was found between the intervention group and the control group. One study [54] investigated the effect of an educational campaign and Vaccine Day. Combining both was the most effective strategy for increasing the vaccine coverage (53% in the intervention group compared to 27% in the control group). Vaccine Day alone was also effective (46% coverage), while the educational campaign alone was not effective in improving coverage levels (34% coverage). The third study [57] found an increase in the vaccination rates at residential care facilities after vaccination was provided at no charge through onsite clinics and the introduction of a mandatory vaccination policy from 57% for the 2011/2012 flu season to 75% for the 2012/2013 flu season (difference in the proportion regarding 2011/2012 = 0.18, 95% CI: 0.18–0.19, *p* < 0.001). One study of moderate quality [75] found that nursing homes with formal education programmes and easy access to vaccination have a 10% higher staff vaccination rate (*p* < 0.001), and higher odds of achieving a 60% vaccination rate (OR = 1.77, *p* = 0.01).

### 3.5. Studies with Interventions Related to Three Areas

Ten studies [51,53,60,64,66,67,69,70,71,76] assessed the impact on vaccination rates of interventions related to three areas taken together. However, only two studies [51,53] were of strong quality. The first [51] evaluate the effect of a vaccination campaign, which consisted of free vaccination on site, educational media, reminder for staff, and monitoring progresses. Vaccination rates increased from 27.6% at baseline to 33.7% after the intervention while, in the control group, vaccination rates decreased from 24.2% to 22.9%. The second study [53] evaluated the introduction of policy for influenza vaccination. It was performed by training lead nurses in each of the intervention homes to promote the influenza vaccine to staff and to arrange for three vaccination sessions within the homes, including at least one session during a night shift, to maximize the uptake. In 2003–2004, the vaccination coverage in full-time staff was 48.2% at intervention homes and 5.9% at control homes, while the uptake rates were 43.2% and 3.5%, respectively, from 2004–2005.

Two studies [63,74] of moderate quality examined the effect of all three areas of interventions. The first [63] reported that the vaccination rate among HCWs in the study period (2013–2017) ranged from 90–98.4% for HCWs working where employer vaccination was a requirement to be employed, from 67.3–80.4% for HCWs working where on-site vaccination was offered, from 54.1–83.0% for HCWs working where on-site vaccination was provided for only one day, and from 58.5–71.7% for HCWs working where other vaccination promotions were performed. The vaccination coverage was the lowest (38.6–44.3%) among healthcare providers working in locations where employers do not require vaccination, provide on-site vaccination at no cost, or promote vaccination. The second [74] found that influenza vaccination was independently associated with an employer vaccination requirement (prevalence ratio (PR) (95% confidence interval) = 1.28 (1.11, 1.47)), which offered free onsite vaccination (PR = 1.20 (1.04, 1.39)), and employers publicizing the vaccination coverage level to employees (PR = 1.24 (1.09, 1.41)). No significant association was found between being informed of the risks and benefits of vaccination and being vaccinated. However, vaccination was most highly associated with a combination of three or more workplace interventions.

## 4. Discussion

### 4.1. General Issue

The influenza vaccination uptake among HCWs at LTCFs is a relevant issue due to its potential risk of transmission to fragile residents and the associated mortality and morbidity. Moreover, the tendency to remain absent during influenza outbreaks can affect the quality of care [77,78]. Although the World Health Organization (WHO) states that ‘influenza vaccination should be offered to all staff who will potentially have contact with LTCF residents’ [79], the influenza vaccination uptake among the LTCF staff varies between countries and facilities, with vaccination coverages generally lower than those of the residents and those of the staff working in hospital settings [77,80,81]. According to WHO guidelines, many interventions should be performed to improve influenza vaccination acceptance and coverage in this setting, but it is still hard to establish which of them has the higher impact on the vaccination uptake.

### 4.2. Main Considerations about the Results

According to our selection criteria, 27 studies published from 1993–2019 addressed the research question. This scientific production highlights the relative lack of research studies performed in this setting, even though the included studies describe interventions conducted in many different countries. No study was performed in a developing country. The applied interventions can greatly vary and be classified, as previously reported, in those aimed at improving access to vaccination, those dedicated to eliminating individual barriers, and those focused on introducing policy interventions directed to mandate vaccination. Most of the studies reported multiple interventions, while the others describe the effect of a single intervention or of several of them. In 26 out of 27 studies, an increase in the influenza vaccination coverage or a positive association between intervention and vaccination was observed with a large variability. The results of this review suggest that healthcare system-based interventions improve the influenza vaccination uptake among HCWs at LTCFs, which generally results in an increase of vaccination adherence, particularly when vaccine administration is performed after the assessment of specific needs and barriers. However, according to the state of the art assessed in this review, vaccine uptake in healthcare workers of LTCFs shows a miscellaneous pattern, reflecting the same heterogeneity of the different organization models of each healthcare structure in each country. The level of vaccination varied greatly in the published studies and it is not possible to clearly identify which interventions are more useful in increasing the uptake of influenza vaccination. However, there are some exceptions. As expected, the most relevant single intervention is the introduction of a mandatory vaccination policy. Two studies of moderate quality [42,74] after the implementation of mandatory vaccination report the highest vaccination coverages (ranging between 90% and 98%) among all studies considered in our review. In contrast, educational interventions are not always useful. In two studies of strong quality [52,54], educational campaign failed to increase vaccination uptake in HCWs. One study of moderate quality [74] also did not find any significant association between being informed of the risks and benefits of vaccination and being vaccinated. Our study suggests that better results in the vaccination uptake in HCWs were obtained by combining more interventions in different areas. Two studies [54,74] analyse the effect of both single interventions and a combination of them. In both studies, a combination of multiple interventions was more effective than any single intervention outside of employer vaccination requirements at increasing vaccination coverage. In fact, educational campaigns related to Vaccine Day is an effective strategy to increase the vaccine coverage among healthcare workers of LTCFs, especially when lead nurses involved in the programme are trained to promote the influenza vaccination for the staff. Educational campaigns can also be effective when they are performed after evaluating the barriers to influenza vaccination among healthcare workers.

LTCFs cover different types of healthcare settings, varying for context, residents, and staff characteristics, services provided to the residents, and size. They can also differ among countries and even within the same country, primarily due to distinctive regulations at the national or regional level and to peculiar needs of each geographical area. In fact, long-term care policies, including those regarding residential facilities, differ considerably between countries, due to nation’s structure and organization, history, culture, or even economic performance [82,83,84,85,86]. This diversity partially explains the variety in the vaccination uptake among the staff in the studies included in this review (from 0%–100%), the interventions that have been implemented, and the effect of the interventions in improving vaccination. For example, Russel [71] assesses the effect of mandatory vaccination, which is not legally enforceable in all countries. Most researchers working on the topic report interventions based on national or regional policies aimed at increasing vaccine uptake. In some cases, they have conducted studies or implemented surveillance activities because of national or regional recommendations [52,53,57,59,61,63,64,65,66,67,69,71,72,73,74,75,76]. Although these policies have the same aim (i.e., to improve HCWs’ vaccination uptake), the implemented interventions differed. They are tailored to specific barriers and needs only in three cases [54,56,60].

Two of the included studies reported the effect of interventions proposed as part of the quality improvement process [62,76]. They were both conducted in the USA, where the quality of care at LTCFs has been a topic of great interest for a longer time than in other countries [87,88]. The quality of care in LTCFs has attracted plenty of interest in recent years and it is now one of the most challenging issues for policymakers. In this setting, poor quality of care represents an issue of public concern and discussions are taking place to address this topic [82,89,90]. Due to the impact of influenza outbreaks on the health of residents and the organization of the healthcare provided, the influenza vaccination rate among staff should be considered a patient safety issue, which is to be monitored using a specific indicator of quality of care [91], as it has already been done for the vaccination rate among residents [92,93].

Lastly, HCWs and healthcare organizations should promote strategies and educational interventions to increase the awareness of knowledge, attitudes, and behaviours toward flu vaccination and the adoption of preventive measures in such populations and settings [94,95].

### 4.3. Strengths and Weaknesses

Based on our knowledge, this is the first systematic review of the utility of healthcare system-based interventions in improving the influenza vaccination uptake in HCWs at LTCFs. Moreover, the results summarize a great number of different interventions currently described in the primary studies performed in this setting. About half of the included research papers have a study design that considers both intervention and control groups. This type of study is the best to test the etiologic hypothesis [96], even though only seven studies were evaluated as ‘strong’ for their quality. Thirteen studies have a cross-sectional design or are interrupted time-series analysis. Because of the nature of the study design, it is not possible to work out whether an association between exposure (proposed interventions) and outcome (vaccination rate) underlies a cause-effect relationship [97]. Moreover, as a limitation of our review, the studies retrieved included many types of different interventions that do not allow a conclusive quantitative synthesis of the utility of each intervention.

Lastly, the publication bias may have led to an overestimation of the effect of healthcare system-based interventions. We found only one study [52] that did not report an increase in vaccination coverage after implementing an intervention. A recent review [98] has shown that there is strong evidence of an association between significant results and publication. Specifically, studies that report positive or significant results are more likely to be published and outcomes that are statistically significant have higher odds of being fully reported.

## 5. Conclusions

According to the results of this review, healthcare system-based interventions can be useful to improve the influenza vaccination uptake among HCWs at LTCFs. The heterogeneity of LTCFs, and the fragmentation of the interventions proposed in the studies prevent the identification of the most effective strategies in improving the vaccination rate. As expected, mandatory vaccination seems to be the most useful intervention to increase the vaccination uptake in HCWs. Our study suggests that better results in the vaccination uptake in HCWs were obtained by combining more interventions in different areas. Educational campaigns alone could not have an impact on vaccination coverage. Further studies, concerning long-term intervention programmes, will be necessary to ascertain the effectiveness of preventive measures at LTCFs.

## Figures and Tables

**Table 1:** Synthesis of the interventions performed in the studies, by area of intervention.

Area	Interventions	Code
Interventions to improve the access to vaccination	Vaccination available at work, in any locations	1a
	Offering vaccine during new hire orientation	1b
	Temporal access (vaccination offered during day and night shifts)	1c
	Continuing to provide vaccines throughout the season	1d
	Holding vaccination kick-off events	1e
	Free vaccination offered	1f
	Provide vaccination at reduced cost	1g
Interventions to eliminate individual barriers (attitudes/opinions)	Identification of the individual and context barriers to vaccination	2a
	Improve vaccination confidence for directors	2b
	Providing in-service educational seminars or educational sessions for staff	2c
	Displaying educational written materials (posters, leaflets,…)	2d
	Offering incentives (treats, raffle tickets) to vaccinated health care workers	2e
	Providing additional education to staff who declined the vaccine	2f
	Individual counselling sessions	2g
	Facilities provide reminders to staff to be immunized	2h
Policy/leadership interventions directed to lead to vaccination	Introduce a policy on immunization	3a
	Mandatory vaccination policy	3b
	Facility recommends influenza immunization for staff	3c
	Requiring completion of declining forms	3d
	Consider vaccination rate as a target for quality improvement	3e
	Identified lead persons to arrange for vaccination sessions and vaccination promotion	3f
	Facilitation support by experts	3g
	Discussing proposed policies and goals with researchers	3h
	Continual performance feedback and shared learning	3j
	Communicating with staff about vaccination goal	3k
	Sharing vaccination rates with staff	3l
	Communicating with staff about new policies on immunization	3m
	Requiring vaccine receipt or masking throughout the season	3n
	Discussing vaccination policy during new hire orientation	3o

## Supplementary Materials

The following are available online at https://www.mdpi.com/2076-393X/8/2/165/s1. Box S1: Search strings used for each database (Pubmed; Web of science; Cinhal; Health evidence; Cochrane library). Table S1: Synthesis of the studies involving intervention and control groups and Quality Assessment (EPHPP) of each study. Table S2: Synthesis of the studies not involving intervention and control groups and Quality Assessment (EPHPP) of each study.
