# Supplementary material for: Utility of Healthcare System-Based Interventions in Improving the Uptake of Influenza Vaccination in Healthcare Workers at Long-Term Care Facilities: A Systematic Review
Source: Vaccines (Basel). 2020 Apr 5;8(2):165. doi: 10.3390/vaccines8020165 (PMC7348755; doi:10.3390/vaccines8020165)
Supplement: Supplementary file 1 [file vaccines-08-00165-s001.pdf]

vaccines

MDPI

Supplementary Materials

Box S1. Search strings used for each database (Pubmed; Web of science; Cinhal; Health evidence; Cochrane library).

**Pubmed**		
P	In health care workers of long-term facilities	((((worker*) OR (professional*) OR personnel OR (provider*) OR (employee*) OR workforce) AND (health OR healthcare OR health-care OR medical)) OR (medical staff) OR (nursing staff) OR hcw OR (physician*) OR (nurse*) OR (doctor*) OR (practitioner*) OR (groups, occupational[MeSH Terms]) OR (health personnel[MeSH Terms])) AND ((“long-term facilit*”) OR (“rest home*”) OR (geriatric) OR (“long term facilit*”) OR (clinic) OR (clinics) OR (“nursing home*”) OR “long-term care” OR “long term care” OR (nursing homes[MeSH Terms]) OR (long-term care[MeSH Terms]))
I	Are health care system-based interventions useful	((((encourag*) OR (increas*) OR (support*) OR (improv*) OR (rais*) OR (promot*) OR (improv*) OR (enhanc*)) AND (access OR reward* OR awareness)) OR ((multi-faceted OR evidence-based OR “healthcare system” OR health-care system) AND (intervention*)) OR (program*) OR polic* OR (strateg*) OR (reminder*) OR (incentive*) OR (mandat*) OR (“lead advocate*”) OR (obligat*) OR “quality improvement” OR “vaccine day” OR (educat*))
C	Compared to no intervention	
O	In increasing the uptake of influenza vaccination?	(((((influenza[MeSH Terms]) OR (influenza) OR (flu)) AND ((vaccine*) OR (vaccina*) OR (immuni*) OR (vaccines[MeSH Terms])) OR (influenza vaccines[MeSH Terms]))) AND (uptake OR (increas*) OR (effect*) OR coverage OR (cover*) OR (acceptanc*) OR (complianc*) OR adoption OR (rate*) OR (maximi*) OR access))
**Web of Science**		
P	In health care workers of long-term facilities	(((((influenza) OR (flu)) AND ((vaccine*) OR (vaccina*) OR (immuni*)))) AND (uptake OR (increas*) OR coverage OR (cover*) OR (acceptanc*) OR (complianc*) OR adoption OR (rate*) OR (maximi*) OR access))
I	are health care system-based interventions useful	((((worker*) OR (professional*) OR personnel OR (provider*) OR (employee*) OR workforce) AND (health OR healthcare OR health-care OR medical)) OR (medical staff) OR (nursing staff) OR hcw OR (physician*) OR (nurse*) OR (doctor*) OR (practitioner*)) AND ((“long-term facilit*”) OR (“rest home*”) OR (geriatric) OR (“long term facilit*”) OR (“nursing home*”) OR “long-term care” OR “long term care”)
C	Compared to no intervention	
O	In increasing the uptake of influenza vaccination?	((((encourag*) OR (increas*) OR (support*) OR (improv*) OR (rais*) OR (promot*) OR (improv*) OR (enhanc*)) AND (access OR reward* OR awareness)) OR ((multi-faceted OR evidence-based OR “healthcare system” OR health-care system) AND (intervention*)) OR (program*) OR (polic*) OR (strateg*) OR (reminder*) OR (incentive*) OR (mandat*) OR (“lead advocate*”) OR (obligat*) OR “quality improvement” OR “vaccine day” OR (educat*))
**Cinhal; Health Evidence; Cochrane Library**		
(influenza or flu) AND (workers or employees or staff or personal or doctor* or nurse*) AND (long-term care or nursing home) AND (vaccine or vaccines or vaccinations or vaccination or immunization or immunizations)		

Table S1. Synthesis of the studies involving intervention and control groups and Quality Assessment (EPHPP) of each study.

Author, Year[references number]	Country	Study Design	Setting	Population		Interventions	Outcome		Quality Assessment (EPHPP)
				Intervention Arm/Group	Control Arm/Group		Intervention Arm/Group	Control Arm/Group	
Black, 2017[63]	USA	Trend in vaccination rate, by interventions	LTCs	-	-	Employer vaccination requirement, on-site vaccination >1day, on-site vaccination 1day, other vaccination promotion	Vaccination rate among HCWs in the study period (2013–2017): ranged from 90–98.4% for HCWs working where employer vaccination was a requirement to be employed, from 67.3–80.4% for HCWs working where on-site vaccination >1day, from 54.1–83.0% for HCWs working where on-site vaccination 1day, from 58.5–71.7% for HCWs working where other vaccination promotion was performed	Vaccination rate among HCWs in the study period (2013–2017): ranged from 36.4–44.3%	Moderate
Borgey, 2019[51]	France	cluster-randomized controlled trial	NHs	11 NHs, 469 HCWs	15 NHs, 840 HCWs	Influenza vaccination campaign	Vaccination rates from 27.6% at baseline to 33.7% after the intervention	Vaccination rates from 24.2% at baseline to 22.9%	Strong
Chambers, 2015[50]	Canada	Randomized controlled trial with pre-post evaluation	Continuing care organizations	2 Continuing care organizations	4 Continuing care organizations	The Intervention group received the Guide "Successful Influenza Immunization Programs for Healthcare Personnel: A Guide for Program Planners", facilitation support through workshops for managers and ongoing support.	Vaccination rate (facility-level): from 65% at baseline (2008–2009), to 87% (2011–2012)	Vaccination rate (facility-level): from 72–92% at baseline (2008–2009) to 67–80% (2011–2012)	Strong
Dey, 2001[52]	UK	Randomized controlled trial	NHs	17 NHs, 768 HCWs	17 NHs, 1,364 HCWs	The NHs in the intervention group were visited by a public health nurse, who raised awareness of the campaign, emphasized the efficacy and safety of vaccination, outlined the possible side effects and contraindications, discussed the impact of influenza on absenteeism, attempt to allay anxiety and to correct misconceptions, disseminated	Vaccination rate: 10.2% HCWs (the difference was not significant).	Vaccination rate: 5.6% (the difference was not significant).	Strong

						promotional materials and informed staff where they could obtain vaccination free of charge			
Hayward, 2006[53]	UK (England)	Pair matched cluster randomised controlled trial.	Care homes	22 Care Homes, 1610 staff unit in 2003–2004, 1726 staffs in 2004–2005	22 Care Homes, 1674 staff unit in 2003–2004, 1766 staffs in 2004–2005	Policy for influenza vaccination. Lead nurses in each of the intervention homes were trained to promote influenza vaccine to staff. They were encouraged to act as advocates for vaccination and to use word of mouth, leaflets, and posters to promote vaccination. A letter was sent to the staff explaining the study and the potential benefits of influenza vaccination. The lead nurse liaised with a local occupational health service to arrange for three vaccination sessions within the homes, including at least one session during a night shift to maximise uptake.	Vaccination rate at facility-level: 35.4% in the first year (2003–2004), 30.5% in the second year (2004–2005)	Vaccination rate at facility-level: 5% in the first year (2003–2004), 3.8% in the second year (2004–2005)	Strong
Kimura, 2007[54]	USA (California)	Randomized controlled trial, with pre-post evaluation	LTCFs	14 LTCFs in group B, 14 LTCFs in group C, 14 LTCFs in group C	25 LTCFs	After having examined the barriers to influenza vaccination among HCWs the following interventions were performed: an educational campaign (group B), a Vaccine Day, a well-publicized day for free influenza vaccination of all employees at the worksite (group C), or both (group D)	Vaccination rate varied for group B from 29% (baseline) to 34%, for group C from 35% (baseline) to 34%, for group D from 39% (baseline) to 53%. An educational campaign combined to Vaccine Day (group D) was the most effective strategy in increasing vaccine coverage (53% in the intervention group compared to 27% in the control group). Vaccine Day alone was also effective (46% coverage), while the educational campaign alone was not effective in improving coverage levels (34% coverage).	Vaccination rate: from 31% (baseline) to 28%	Strong
Ksienski, 2014[57]	USA (British	Pre-post survey using	Residential care Facilities	332 Residential	-	On August 31, 2012, the BC Ministry of Health announced	Vaccination rates at residential care facilities: 75% for the	Vaccination rates at residential care facilities: 57% for the	Strong

	Columbia-BC)	BC Centre for Disease Control data		care Facilities; 36,620 HCWs		implementation of the province-wide Influenza Prevention Policy, that requires all HCWs to be vaccinated annually against influenza. Influenza vaccination is provided at no charge through onsite clinics and can also be obtained from an individual's general practitioner or local pharmacy. HCWs are obligated to report their vaccination status annually to Infection Control. Once vaccinated, a HCW must place a green dot on his or her identification tag. HCWs who witness any colleagues violating the Policy are required to report the incident to their supervisor. Non-compliance would result in remedial action; continued violation of the Policy could ultimately result in termination of employment, contract cancellation or revocation of faculty privileges.	2012/2013 flu season (difference in proportion with regard to 2011/2012 = 0.18, 95% CI: 0.18–0.19, $p < 0.001$).	2011/2012 flu season (difference in proportion with regard to 2012/13 = 0.18, 95% CI: 0.18–0.19, $p < 0.001$).	
Lemaitre, 2009[55]	France	Cluster randomised controlled trial	NHs in which the staff influenza vaccination coverage rate was less than 40% during the 2005/06 winter season	20 NHs; 989 staff unit	20 NHs; 1015 staff unit	Promotional campaign based on posters, leaflets, and an information meeting with the study team. Influenza vaccination was further recommended during face-to-face interviews. The study team individually met all administrative staff, technicians, and caregivers to invite them to participate, and volunteers were vaccinated at the end of the interview. In the control arm, only routine	Vaccination rate calculated at NH-level (questionnaire sent to each staff unit): 69.9% (range 48.4–89.5%).	Vaccination rate calculated at NH-level (questionnaire sent to each staff unit): 31.8% (range 0–69.0%)	Strong

						information on influenza vaccination was provided.			
Looijmans-van den Akker, 2010[56]	The Netherlands	Cluster randomised controlled trial	NHs. NHs that did not intend to offer routine influenza vaccination to their HCWs were ineligible	18 NHs; 193 average number of HCWs	17 NHs; 209 average number of HCWs	Multi-faced implementation program, developed applying the intervention mapping method based on the outcome of previous studies. It consisted of three main components: the NHs received a script of the program, all required materials and background information (component A), a plenary 1 h information meeting (organized twice in each NHs) by a specialised nurse of the local municipal health centre (component B) and appointment of preferably a physician as local program coordinator (component C). In the control arm, usual program were performed	Vaccination rate calculated at NH-level (uptake registered on site): from 20% (baseline; range: 4-40%) to 25% (after intervention; range: 4.4–80.6%).	Vaccination rate calculated at NH-level (uptake registered on site): from 21% (baseline; range: 1.9–50%) to 16% (in the year of the intervention; range: 0.4–35.8%)	Weak
Nace, 2011[58]	USA (Pennsylvania)	Pre-post survey with control group	LTCFs	3 LTCFs; 610 staffs	3 LTCFs; 658 staffs	Six facilities assembled immunization teams of at least 4 staff members. The teams assessed their facility's baseline immunization program, calculated immunization rates of HCWs, and planned and implemented changes to their immunization programs. Three of the six facilities participate to a collaborative training program, based on the Institute for Healthcare Improvement model, provided didactic education, a review of baseline immunization rates, training in barrier identification, and goal setting. Teams defined	Vaccination rate calculated at LTCF-level: improved 10.9% (range: 0.4–21.9%), from 39.2% (2002) to 50.1% (2003)	Vaccination rate calculated at LTCF-level: decreased 3.5% (two decreased of 10.6% and 16.7% respectively, one increased 16.9%), from 29.3–25.8%	Moderate

						program intervention, specified process and outcome measurements, and set completion time frames.			
Nace, 2012[59]	USA (Pennsylvania)	Pre-post survey	LTCFs	14 LCTF among 16 served by LTC pharmacy; for 4 LTCF pre-post evaluation	-	Facilities had to specifically transfer the location of HCW immunization policies and procedures from the domain of nursing (Nursing Policy and Procedure Manual) to that of the LTC pharmacy (Pharmacy Policy and Procedure Manual). In addition, facilities had to agree to work collaboratively with the pharmacy to address issues of vaccine supply and ordering, revision of HCW consent processes, vaccine administration and record keeping, data collection, and staff feedback. The LTC pharmacy implemented a HCW immunization program at each facility. Facilities were expected to designate an immunization champion, participate in educational activities and ensure leadership	Vaccination rate calculated at LTCF-level: on the 4 LTCFs, about 60%-80%.	Vaccination rate calculated at LTCF-level: on the 4 LTCFs, 40%-50% at baseline	Weak
Ofstead, 2017[60]	USA (Illinois)	Pre-post survey	LTCFs	4 LCTF; 518 nurses	-	The theoretical basis for the study incorporated elements of the Health Belief Model (HBM) and an ecological model. After a baseline assessment, customized interventions were performed, including educational programming, vaccination tracking mechanisms, and worksheets to assist with program implementation (e.g., goal-setting, policy	Vaccination rate at LTCF-level (administration data): 85% (2015)	Vaccination rate at LTCF-level (administration data): 50% (2014)	Weak

						development, and vaccination kick-off events)			
Sand, 2007[61]	USA	Pre-post survey	LTCFs	13 LTCF	-	The Quality Improvement (QI) teams first evaluated their current immunization program, its performance, and known barriers to improvement. Then, the QI created the strategy to improve vaccination rate. The strategies addressed the following barriers: vaccine access; education; reverse consent (declination consent); leadership involvement; incentives.	Vaccination rate at LTCF-level (LTCF-level): 11 of the 13 LTCFs that used QI saw improvements in their staff immunization rates; 10 improved more than 10%, and seven improved to more than 55%. *	-	Weak
Thomas, 1993[62]	USA (North Carolina)	Pre-post survey	LTC	1 LTC; 195 staff unit (60 not HCWs)	-	Educational intervention with individual encouragement and answering of questions. The educational intervention culminated in a 1-day Staff Vaccination Fair in which influenza vaccine was offered for free. In the year after the intervention, Vaccination Fair was repeated	Vaccination rate among staff: 46% during first Vaccination Fair and 54% during second Vaccination Fair. Physician staff, administrative staff and maintenance staff achieved the highest rates (100%, 92% and 85% respectively)	Vaccination rate among staff: 8% in the pre-intervention year	Weak

*data in the main text are different from those reported in the tables; EPHPP: Effective Public Health Practice Project’s; LCT: Long-Term Care; LCTF: Long-Term Care Facility; NH: Nursing Home; HCW: health care worker.

Table S2. Synthesis of the studies not involving intervention and control groups and Quality Assessment (EPHPP) of each study.

Author, Year [references number]	Country	Study Design	Setting, Population	Interventions	Outcome	Main Findings	Quality Assessment (EPHPP)
Apenteng, 2014[64]	USA	Cross-sectional (facility-level). Data from the 2010 National Survey of Residential Care Facilities were used.	2303 LTCFs	Recommending vaccination to employees; providing vaccinations on site; providing vaccinations to employees at no cost; requiring vaccination (or contraindication) as a condition of employment; providing vaccines at reduced cost; providing incentives for employee vaccination; and having a patient restriction policy for sick employees	Vaccination rate: 100% in the 24.1% of the LTCFs; 81-99% in the 18.7% of the LTCFs; 61–80% in the 13.1% of the LTCFs;1–60% in the 36.5% of the LTCFs; none in the 7.5% of the LTCFs	Whereas a higher proportion of facilities achieving a 100% influenza vaccination rate required proof of vaccination as a condition for employment, a higher proportion of facilities with employee influenza vaccination rates between 81% and 99% recommended vaccinations, provided vaccinations on site and for free, incentivized their staff for vaccination, and had a patient restriction policy in place for employees who developed flu-like symptoms. After adjusting for confounding, organizational factors, facilities achieving high employee influenza vaccination were more likely to recommend vaccination to staff (OR, 1.58; $p < 0.001$), offer vaccinations on site (OR,1.46; $p < 0.01$), offer vaccinations for free (OR, 1.76; $p < 0.001$), provide staff with incentives for vaccination (OR, 1.53; $p < 0.05$), and require proof of vaccination or contraindication as a condition for employment (OR,1.93; $p < 0.001$). Providing vaccines at a reduced cost and having a patient restriction policy for sick employees were not independently associated with employee influenza vaccination rates in the study sample.	Weak
Bentele, 2014[65]	Norway	Cross-sectional (facility-level)	354 NHs (of 910 invited); 28,237 HCWs	Financing influenza vaccination for HCWs, vaccination promotion (vaccination campaign).	Vaccination rate: the median vaccination rate was 0; 214 (60%) NHs reported a vaccination rate equal to zero	No significant difference was observed in vaccination rate between NHs in which vaccination was free with respect to those in which HCWs have to pay for it. The median vaccination rate for HCWs in the NHs having an annual vaccination campaign was 0% (range 0–53), compared to when they did not have an annual vaccination campaign 0% (range 0–12); the distributions in the two groups differed significantly ($p = 0.006$).	Weak

Borlaug, 2007[66]	USA (Wisconsin)	Cross-sectional (facility-level)	268 NHs (of 405 invited)	Vaccination provided for free, education to promote vaccination, declination form required	Vaccination rate (overall): the median vaccination rate was 50% (range: 5–100%)	Median rates of influenza vaccination among HCWs were significantly higher at NHs that offered influenza vaccination for free, compared with those that did not (50% vs. 30%); at those that provided education, compared with those that did not (50% vs. 36%); and at those that required signed declination forms, compared with those that did not (62% vs. 50%).	Weak
Campbell, 2014[75]	USA (Oregon)	Interrupted time series (2009–2012). Data were gathered from Office for Oregon Health Policy and Research, the Online Survey, Certification, and Reporting and from the 2010 Area Health Resource File.	137 NHs	Vaccination promotion and education program	Vaccination rate: ranging from 54–52% in 2009–2011	NHs with formal education programs have 10% higher staff vaccination rate ($p < 0.001$), and higher odds of achieving 60% vaccination rate (OR=1.77, $p = 0.01$).	Moderate
Halliday, 2003[67]	Australia (Australian Capital Territory)	Cross-sectional	19 residential aged care facilities; 587 staff	Access to influenza vaccine at the facility,<br>The facility: organises vaccination for staff, provides reminders to staff to be immunised, documents influenza vaccination status of staff, provide staff with information on the need to be immunised need to be immunised, has a policy of staff vaccination, has a policy of staff vaccination for influenza, recommends that staff be immunised for influenza.	Vaccination rate: median of 25% (range: 9–57%)	Uptake of the vaccine was associated with particular policies and practices of facilities such as: organisation of vaccination for staff (OR 2.52, CI 1.38–4.59, $p < 0.001$); provision of reminders to staff (OR 2.73, CI 1.70–4.06, $p < 0.001$); and provision of information on the need for vaccination (OR 1.94, CI 1.03–3.67, $p < 0.001$). Other factors significantly associated with influenza vaccine uptake included: vaccination arranged by the workplace (OR 4.02, CI 2.66-6.07, $p < 0.001$), and awareness of recommendations for staff employed in aged care facilities (OR 3.66, CI 2.28–5.85, $p < 0.001$).	Weak
Hauri, 2006[68]	Germany (Hesse)	Two cross-sectional surveys	50 NHs (directors); 846 staffs, from 36 NHs	Vaccination offered free of charge to employees, vaccination offered at work site	Vaccination rate: 22% overall, ranged from 0–29% in 30 facilities and from 50–69% in 6 facilities	The distribution of the facilities according to vaccination rate (low for 0–29%, high for 50–69%) was significantly associated with "vaccination offered free of charge to employees" but not with "vaccination offered at work site"	Weak

Henry, 2001[69]	USA (Ontario)	Cross sectional	508 LTCFs	Vaccination policy, specifically educational sessions for staff, providing individual counseling sessions for staff who had concerns about vaccination, holding on-site immunization clinics on day, evening and night shifts, having a written facility policy for influenza prevention and control	Vaccination rate (facility-level): ≥70% for 80.9% of the facilities, <70% for 19.1% of the facilities	Strategies associated with increased staff immunization levels included holding educational sessions for staff, providing individual counseling sessions for staff who had concerns about vaccination, and holding on-site immunization clinics on day, evening and night shifts. Facilities with high staff immunization coverage were significantly more likely to have a written facility policy for influenza prevention and control.	Weak
Nace, 2007[76]	USA (Pennsylvania)	Interrupted time series (1996–2006)	One LTCF, 211–242 staffs	Members of the quality improvement (QI) performed a current condition assessment in which they clarified the current staff immunization process. Next, a formal needs analysis was conducted to determine the barriers to, and drivers of, staff immunization. Interventions were: vaccine planning; staff education; leadership commitment; staff notification; vaccine administration at employee work units, during all shifts, throughout the entire flu season; non-responder notification (refusal consent); data tracking; continual performance feedback and shared learning	Vaccination rate: from 54% in 1996 to 95.5% in 2003	Using data from 1996 and 1997 as baseline, staff immunization rates improved from 54–55% to between 74% and 95% over past 4 years	Weak
McArthur, 1999[70]	Canada	cross-sectional (facility-level)	1270 LTCFs (of 1520 invited)	Using any encouragement to staff to be vaccinated (eg, made available vaccination clinics, reminders at staff meetings, information posters)	Vaccination rate: 3.7% of the LTCFs indicated that greater then 75% of their staff were vaccinated	Facilities that reported using any encouragement to staff to be vaccinated reported significantly higher vaccination rates: vaccination rate greater then 75% was observed in 5.3% of LTCFs reporting intervention vs. 0.9% of those with no interventions. In particular, of the 34 facilities that indicated they had greater than 75% of their staff vaccinated, 27 had identified or made available vaccination clinics and posted the times of these clinics for staff.	Weak
Russell, 2001[71]	Canada (Alberta)	Cross-sectional (facility-level)	53 LTCFs (of 160 invited)	Mandatory vaccination policy; passive access to vaccination (staff given information on who to approach for vaccination and when to get vaccinated); physical access to vaccination (nurse takes vaccination cart to work stations, cafeteria,	Vaccination rate (median): 36.9%	Several policy and program were correlated with staff vaccination rates: self-reported mandatory vaccination policy; passive access to vaccination (staff given information on who to approach for vaccination and when to get vaccinated); physical access to vaccination (nurse takes vaccination cart to work stations,	Weak

				pharmacy, and other locations and administers vaccine); temporal access to vaccination (vaccination was offered to staff during night shift); program included education for staff; number of Media used to publicize vaccination policy; goal with progress reports (Facility regularly posted, in prominent places, both its goal for vaccination of staff and reports of progress toward that goal)		cafeteria, pharmacy, and other locations and administers vaccine); temporal access to vaccination (vaccination was offered to staff during night shift); program included education for staff; number of media used for communication; facility regularly posted, in prominent places, both its goal for vaccination of staff and reports of progress toward that goal.	
Shroufi, 2009[72]	UK (Nottingham)	Cross-sectional (two survey: one at care home-level, the other at staff-level)	43 care homes (of 58 invited) in the care-home level study; 24 care homes (of 24 invitedt) and 169 staff unit (of 219 invited)	Reported presence of a policy on staff influenza immunization in the care homes (general answer in the questionnaire)	Vaccination rate (overall): 20% in the study at care home-level, 17% in the study at staff-level	Among care homes without a policy on staff influenza immunization, vaccination rate was 22% compared with 42% among care homes which did have such a policy ($p = 0.01$). Staff who responded 'yes' to the statement 'this care home's policy states that I should have vaccine' were 2.5 times more likely to be vaccinated compared with those who responded 'no'.	Moderate
Vaux, 2010[73]	France	Cross-sectional (facility-level)	1218 NHs (of 2186 invited)	Training or meetings on influenza vaccination; if free influenza vaccination was offered to HCWs (including both the vaccine and its administration)	Vaccination rate (overall): 36%	The possibility of a free delivery of influenza vaccination for HCWs, the organization of training sessions or staff meetings on influenza and its prevention through vaccination were associated with a higher influenza vaccination uptake among HCWs (RR = 1.89, RR = 1.20, respectively).	Weak
Yue, 2019[74]	USA	Cross-sectional	332 HCWs	employers publicizing the risks and benefits of vaccination, employers sending a personal reminder to be vaccinated, employers offering on-site vaccination, employer requirement for vaccination	employer vaccination requirement (aPR, 95% CI = 1.28 (1.11, 1.47)), being offered on-site vaccination (aPR, 95% CI = 1.20 (1.04, 1.39)), and having an employer who publicized vaccination coverage levels to employees (aPR, 95% CI = 1.24 (1.09, 1.41)).	Healthcare personnel working in LTC facilities with 3 or more workplace interventions of any type were about 1.5 times more likely to be vaccinated compared with those with no workplace interventions (aPR = 1.48 (1.08, 2.02)). Implementing employer vaccination interventions in LTC facilities, including employer vaccination requirements and free on-site influenza vaccination that is actively promoted, could increase influenza vaccination among healthcare personnel.	Moderate

EPHPP: Effective Public Health Practice Project's; LCT: Long-Term Care; LCTF: Long-Term Care Facility; NH: Nursing Home; HCW: health care worker; QI: Quality Improvement; OR: Odds Ratio; RR: Relative R; aPR: adjusted prevalence rati.
